# Celastrol Ameliorates Renal Injury in Spontaneously Hypertensive Rats by Activating the Nrf2/Ho-1 Signaling Pathway to Alleviate Oxidative Stress

**DOI:** 10.3390/ijms27093849

**Published:** 2026-04-26

**Authors:** Yijie Deng, Jichun Wang, Xiping Liu, Xiuwen Wang, Hua Li, Bo Gu, Min Zhang, Renjun Wang, Yi Yang

**Affiliations:** 1 Department of Biotechnology, College of Life Sciences, Jilin Normal University, Siping 136000, China; 13620770162@163.com (Y.D.); 15648822307@163.com (J.W.); 15903495754@163.com (X.L.); liaoshiwxw@163.com (X.W.); reniunwang211@163.com (R.W.); 2 Department of Bioscience, College of Life Sciences, Jilin Normal University, Siping 136000, China; langyue121@163.com (H.L.); 17743110597@163.com (B.G.); zhangmin19880703@163.com (M.Z.)

**Keywords:** Celastrol, hypetensive renal injury, renoprotection, Nrf2

## Abstract

Celastrol (CSL), a natural triterpenoid extracted from *Tripterygium wilfordii*, demonstrates a wide range of biological activities. In this study, we explored whether CSL alleviates kidney damage in spontaneously hypertensive rats (SHRs) through the modulation of the Nrf2/Ho-1 pathway, a crucial target in renal injury models. A total of 40 male SHRs, aged 6–8 weeks, were randomly allocated to four groups: the control group (CON, serving as the healthy control), the spontaneously hypertensive rat group (SHR), the SHR group treated with low-dose CSL (L-CSL + SHR, 0.5 mg/kg/d), and the SHR group treated with high-dose CSL (H-CSL + SHR, 1 mg/kg/d). All drugs were formulated using physiological saline as the solvent and administered via intraperitoneal injection. The control group received an equivalent volume of physiological saline via intraperitoneal injection, and all groups underwent continuous daily administration for 6 weeks. The results indicated that, in comparison with the control group, the serum levels of angiotensin, angiotensin-converting enzyme, and aldosterone in the SHR group were relatively high, and CSL treatment further downregulated these indices. Simultaneously, CSL downregulated pro-inflammatory factors (tumor necrosis factor-α and interleukin-1β) and upregulated interleukin-6. Regarding renal function-related indicators, CSL reduced malondialdehyde levels and enhanced the activities of antioxidant enzymes, such as superoxide dismutase, glutathione peroxidase, and catalase. Moreover, CSL inhibited the overexpression of Keap1. Significantly, the mRNA levels of Nrf2, Nqo1, and Ho-1 in the CSL-treated groups were notably higher than those in the SHR group. These findings suggest that CSL mitigates renal pathological damage in SHR by activating the Nrf2/Ho-1 pathway, offering a potential therapeutic approach for hypertension-induced renal injury.

## 1. Introduction

Hypertensive renal injury is a severe complication of hypertension and significantly accelerates the progression of chronic kidney disease (CKD) [1,2,3]. Spontaneously hypertensive rats (SHRs) have been widely employed as an animal model to study hypertensive renal injury. The results of studies on SHRs have demonstrated that prolonged hypertension leads to a series of pathological changes in the kidneys [4]. For example, elevated blood pressure in SHRs can cause renal arteriolar sclerosis, glomerular hypertrophy, and tubulointerstitial fibrosis [2,5]. These structural changes are accompanied by functional impairments, such as a reduced glomerular filtration rate (GFR) and abnormal urinary protein excretion, both of which are hallmarks of hypertensive renal injury [6]. Oxidative stress arises from an imbalance between the excessive production of free radicals such as reactive oxygen species (ROS) and insufficient antioxidant defense capacity in the body; the resulting lipid peroxidation, protein modification, and DNA damage can impair cells and tissues and serve as one of the core pathological mechanisms underlying various diseases including hypertension-associated vascular and renal lesions [7]. Specifically, in SHRs, the nicotinamide adenine dinucleotide phosphate (NADPH) oxidase, especially the NOX2 and NOX4 subtypes, is overactivated in vascular endothelial cells and renal tissues, leading to significant accumulation of reactive oxygen species (ROS) [8]. Moreover, the expression of NOX2 and the activity of NADPH oxidase in the aortas of SHRs are increased, which elevates ROS production and consequently induces endothelial dysfunction [9]. Moreover, the excessive accumulation of ROS in SHRs results in decreased endothelial nitric oxide synthase (eNOS) activity and reduced nitric oxide (NO) bioavailability; eplerenone can improve this phenomenon through its antioxidative effect [10]. In addition, research findings have demonstrated that the reduced density of caveolae in the blood vessels of SHRs leads to eNOS uncoupling, which further exacerbates ROS-induced damage to eNOS function and ultimately causes endothelium-dependent vasodilation dysfunction [11]. At the renal level, ROS generated by NOX2/NOX4 in the kidneys of SHRs can activate the TGF-β1/Smad pathway, accelerating the process of renal fibrosis [12].

Pharmacologically active compounds derived from medicinal plants have recently attracted considerable attention due to their potent, unique, and diverse biological activities. Celastrol (CSL), a pentacyclic triterpene isolated from *Tripterygium wilfordii* [13], has been extensively investigated for its therapeutic potential. The results of several studies have demonstrated its anticancer properties, including the promotion of apoptosis and the inhibition of cell invasion, angiogenesis, and proliferation [14,15]. Extracts from *Tripterygium wilfordii* have been investigated in numerous clinical trials due to their broad pharmacological activities. Moreover, randomized clinical trials have been conducted to evaluate CSL in various conditions, such as renal transplantation, rheumatoid arthritis, solid tumors, and diabetic nephropathy, to more effectively assess its therapeutic potential for kidney diseases [16,17]. CSL has been shown to possess antioxidant properties by upregulating the expression and activity of heme oxygenase-1 (Ho-1) and reducing ROS production in vascular smooth muscle cells of hypertensive rats [18]. It has been reported that CSL ameliorates kidney injury induced by a high-fat diet in obese mice by modulating the renal Kelch-like ECH-associated protein 1 (Keap1)/nuclear factor erythroid 2-related factor 2 (Nrf2) pathway to enhance antioxidant capacity [19]. In addition, the results of mechanistic studies have demonstrated that CSL inhibits multiple processes involved in oxidative stress and inflammation, such as the NF-κB signaling pathway [20]. NF-κB, a pleiotropic transcription factor, is considered essential for regulating genes involved in oxidative stress and inflammation, both of which contribute to atherosclerosis [21]. The underlying mechanisms may involve suppression of the NF-κB pathway and reduction in oxidative stress, as demonstrated in related models. In light of the increasing understanding of the complex pathogenesis of hypertensive renal injury and the promising effects of CSL in relevant models, further investigation into its therapeutic potential for hypertensive renal injury is warranted.

## 2. Results

### 2.1. Effects of CSL on Serum Biochemical Indices of SHRs

**Figure 1:**
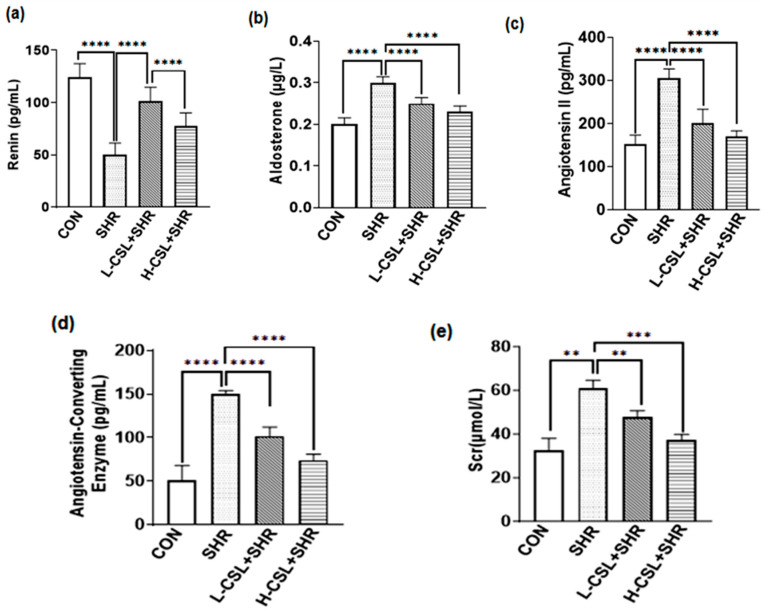
Effect of CSL treatment on serum levels of (**a**) renin, (**b**) angiotensin II, (**c**) aldosterone, (**d**) angiotensin-converting enzyme, and (**e**) serum creatinine. All results are presented as mean ± SD (*n* = 3 per group). The statistical significance is indicated as follows: ** *p* ≤ 0.01, highly significant; *** *p* ≤ 0.001, extremely significant; **** *p* ≤ 0.0001, exceptionally significant.

As shown in Figure 1, serum levels of angiotensin II, aldosterone, angiotensin-converting enzyme, and serum creatinine were significantly elevated in the SHR group compared with those in the CON group (*p* ≤ 0.0001). Compared with this group, both low- and high-dose CSL treatments significantly reduced these biochemical indices (*p* ≤ 0.0001). Notably, the levels of angiotensin II (*p* ≤ 0.0001) and angiotensin-converting enzyme (*p* ≤ 0.0001) in the H-CSL + SHR group were significantly different from those in the L-CSL + SHR group, indicating that catalase CSL inhibited the changes in plasma biochemical indices in SHRs in a dose-dependent manner.

### 2.2. Effects of CSL on the Levels of Inflammatory Factors in SHRs

**Figure 2:**
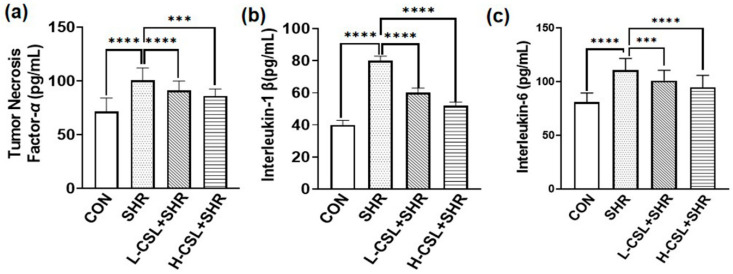
Effect of CSL treatment on serum levels of (**a**) tumor necrosis factor-α, (**b**) interleukin-1β, and (**c**) interleukin-6. All results are presented as mean ± SD (*n* = 3 per group). The statistical significance is indicated as follows: *** *p* ≤ 0.001, extremely significant; **** *p* ≤ 0.0001, exceptionally significant.

As shown in Figure 2, serum levels of tumor necrosis factor-α and interleukin-6 were significantly elevated in the SHR group compared with the CON group (*p* ≤ 0.0001). CSL pretreatment significantly reduced the levels of tumor necrosis factor-α, interleukin-1β, and interleukin-6 (*p* ≤ 0.0001). Moreover, high-dose CSL significantly decreased interleukin-1β levels compared with the low-dose group (*p* ≤ 0.0001). These results suggest that CSL enhances the anti-inflammatory response in SHRs.

### 2.3. Effects of CSL on Oxidative Stress-Related Indices in SHRs

**Figure 3:**
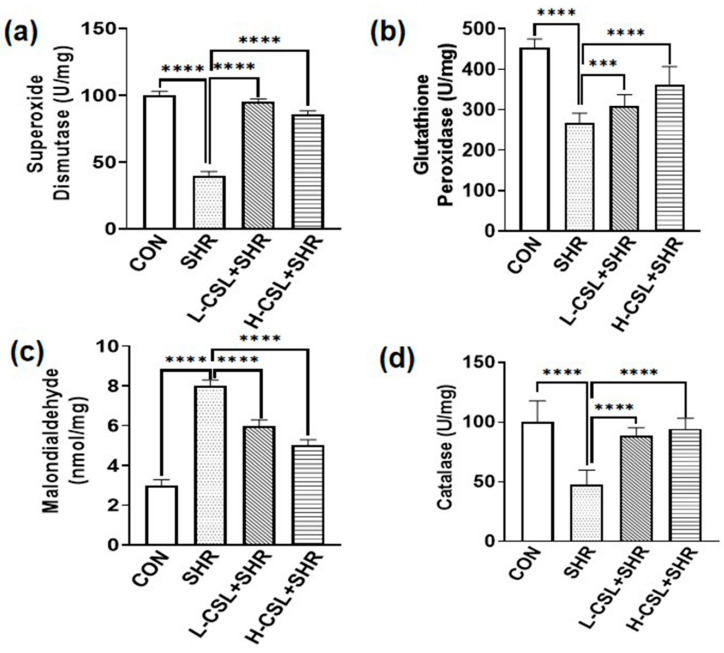
Effect of CSL treatment on kidney antioxidant parameters: (**a**) superoxide dismutase, (**b**) glutathione peroxidase, (**c**) malondialdehyde, and (**d**) catalasealase. All results are presented as mean ± SD (*n* = 3 per group). The statistical significance is indicated as follows: *** *p* ≤ 0.001, extremely significant; **** *p* ≤ 0.0001, exceptionally significant.

As shown in Figure 3, the activities of superoxide dismutase, glutathione peroxidase, and catalase in the kidney tissue of the SHR group were significantly decreased; in comparison, the level of malondialdehyde was significantly increased compared with the CON group (*p* ≤ 0.01). CSL pretreatment significantly enhanced the activities of superoxide dismutase, glutathione peroxidase, and catalase and reduced malondialdehyde levels in the kidneys of SHRs (*p* ≤ 0.0001). Furthermore, the activities of superoxide dismutase, glutathione peroxidase, and catalase in the high-dose CSL group were significantly higher than those in the low-dose group (*p* ≤ 0.0001). These results indicate that catalase CSL improves renal antioxidant capacity in SHRs and exerts a protective effect on the kidney.

### 2.4. Effects of CSL on Renal Pathology in SHRs

**Figure 4:**
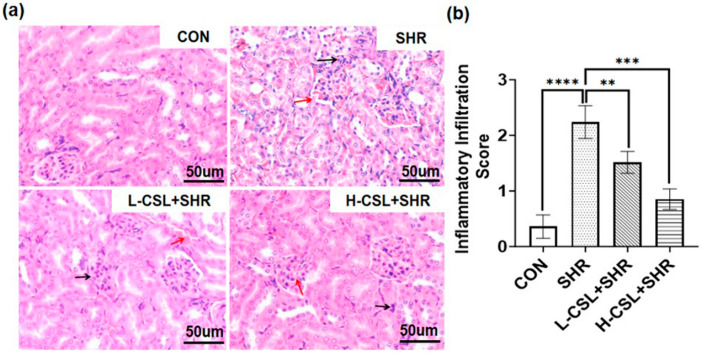
(**a**) Effects of CSL on the morphological changes of kidney tissue in SHRs (400×). (**b**) Comparison of inflammatory infiltration scores among groups. The black arrow points to the inflammatory factors, and the red arrow points to the red blood cells. The statistical significance is indicated as follows: ** *p* ≤ 0.01, highly significant; *** *p* ≤ 0.001, extremely significant; **** *p* ≤ 0.0001, exceptionally significant.

Hematoxylin and eosin (H&E) staining was used to assess pathological changes in renal tissue across the treatment groups, as shown in Figure 4a. In the CON group, glomeruli appeared normal in size and structure, the renal tubular basement membranes were intact, and no diffuse inflammatory cell infiltration or congestion was observed. In the SHR group, the renal tubular basement membrane was thickened, tubular epithelial cells were swollen, and mesangial cell proliferation was evident. Compared with the SHR group, CSL-treated groups exhibited only mild inflammatory cell infiltration, slight thickening of the tubular basement membrane, and marked improvement in congestion, tubular epithelial cell swelling, and mesangial cell proliferation. These findings suggest that CSL attenuates inflammatory cell infiltration and tubular epithelial cell swelling, thereby improving renal inflammatory injury in SHRs, with high-dose CSL demonstrating a more pronounced therapeutic effect than low-dose treatment.

As shown in Figure 4b, the inflammatory infiltration score in the SHR group was remarkably higher than that in the CON group (*p* ≤ 0.0001). Compared with the SHR group, both L-CSL + SHR and H-CSL + SHR groups exhibited significantly reduced scores (*p* ≤ 0.01, *p* ≤ 0.001). Moreover, the score of H-CSL + SHR was lower than that of L-CSL + SHR (*p* ≤ 0.001), indicating that catalase CSL alleviates renal inflammatory infiltration in SHRs in a dose-dependent manner.

### 2.5. Effects of CSL and SFN on the Proliferative Activity of RGECs

**Figure 5:**
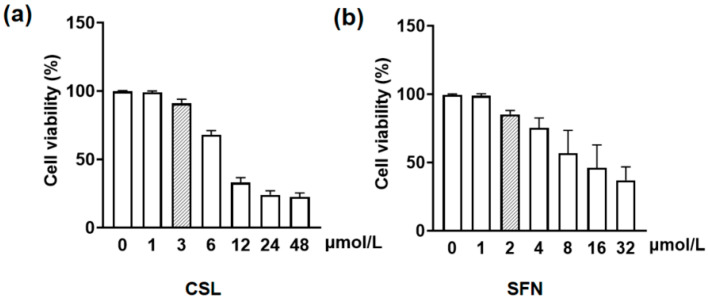
Effects of different concentrations of CSL and SFN on cell viability (**a**). Cell viability (%) after treatment with various concentrations of CSL (0, 1, 3, 6, 12, 24, and 48 μmol/L). (**b**). Cell viability (%) after treatment with various concentrations of SFN (0, 1, 2, 4, 8, 16, and 32 μmol/L).

As shown in Figure 5, compared with the control group, after co-treatment of RGECs with CSL or SFN combined with H_2_O_2_ for 24 h, the proliferative activity of RGECs was significantly increased when the concentration of CSL was above 3 μmol/L and the concentration of SFN was above 2 μmol/L (*p* ≤ 0.01). As a result, these two concentrations were selected for subsequent experiments.

### 2.6. Effects of CSL on the Nrf2/Keap1 Pathway in SHRs: In Vivo Renal Tissue and In Vitro Cell Experiments

**Figure 6:**
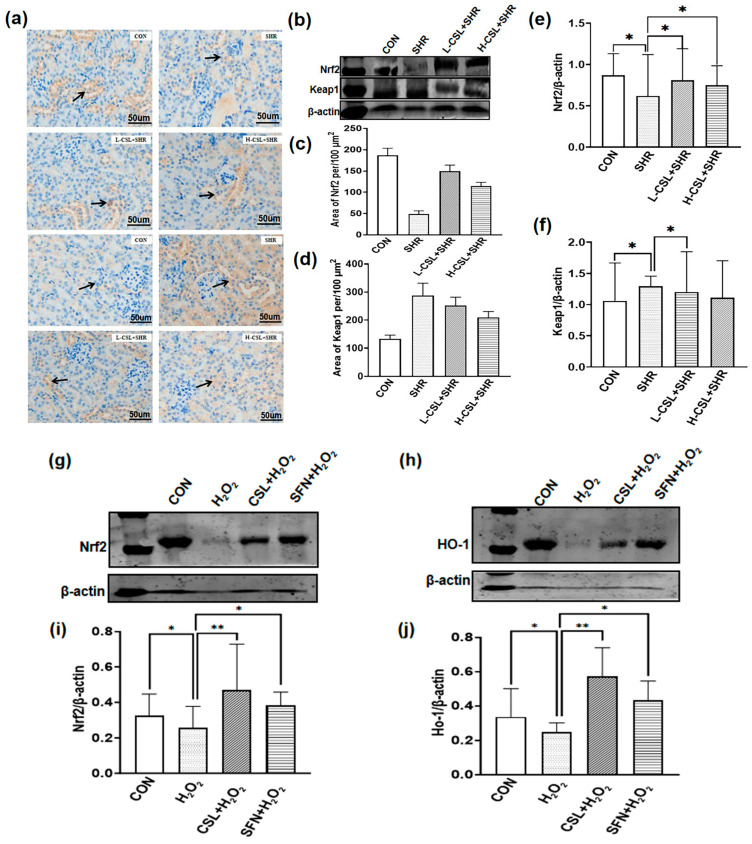
Immunohistochemistry (IHC) and Western blotting (WB) analysis of Nrf2 and Keap1 in kidney tissues. (**a**) Representative IHC images showing Nrf2 and Keap1 protein expression in each group.Arrows mark representative areas of positive protein expression (brown staining). (**b**) Quantification of Keap1 protein area (*n* = 3). (**c**) Quantification of Nrf2 protein area (*n* = 3). (**d**) Representative Western blots of Nrf2 and Keap1 protein levels in kidney tissues, with β-actin as the internal control (*n* = 3). (**e**) Quantitative histological scoring of Nrf2. (**f**) Quantitative histological scoring of Keap1. (**g**,**h**) Western blot bands of Nrf2 and Ho-1; (**i**,**j**) Quantitative analysis of band grayscale. All results are presented as mean ± SD. The statistical significance is indicated as follows: * *p* ≤ 0.05, significant; ** *p* ≤ 0.01, highly significant.

The expression levels of Nrf2 and Keap1 proteins in renal tissues were evaluated using immunohistochemistry and Western blotting, as shown in Figure 6. Compared with the CON group, Nrf2 expression in the kidneys of SHRs was significantly decreased (*p* ≤ 0.05), whereas Keap1 expression was markedly increased (*p* ≤ 0.05). CSL pretreatment significantly upregulated Nrf2 expression and downregulated Keap1 expression in SHR kidneys (*p* ≤ 0.05). Furthermore, Nrf2 and Keap1 expression levels differed significantly between the high- and low-dose CSL groups (*p* ≤ 0.05). These results indicate that Nrf2 and Keap1 are involved in renal tissue injury in SHRs and that CSL modulates their expression.

To further demonstrate that CSL can modulate the Nrf2/Ho-1 pathway, we conducted experiments using the Nrf2 agonist SFN in rats. The results are presented in Figure 6g–j. Compared to the CON group, H_2_O_2_ treatment led to a decrease in the protein expression levels of Nrf2 and Ho-1, whereas the addition of CSL and SFN made it possible to upregulate the expression of both Nrf2 and Ho-1.

### 2.7. Effects of CSL on mRNA Transcription of Genes Related to the Nrf2/Ho-1 Signaling Pathway in the Kidneys of SHRs

**Figure 7:**
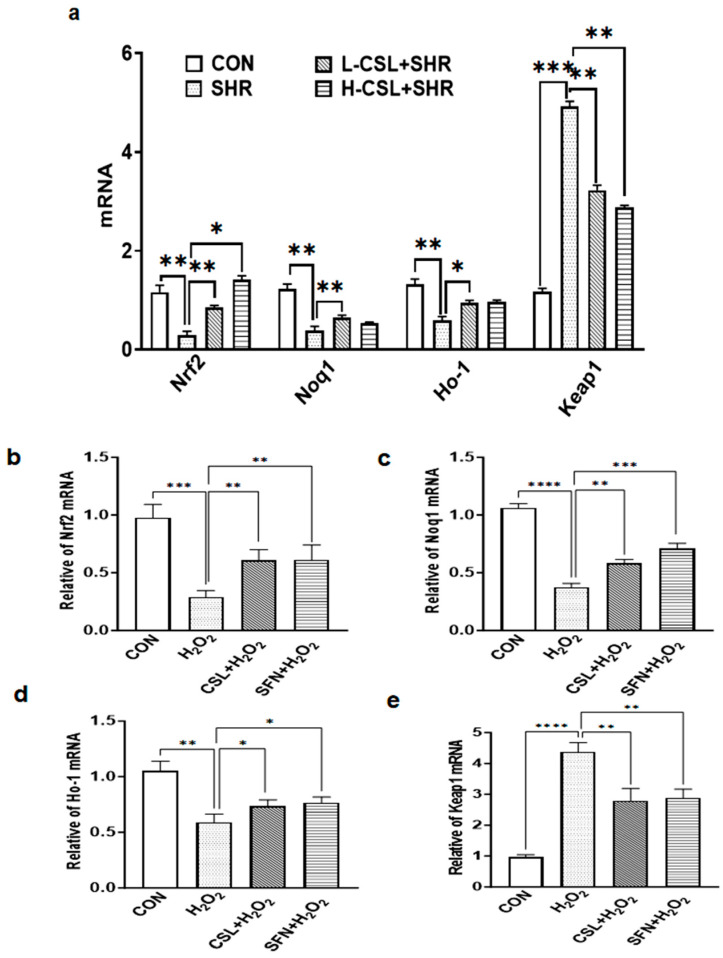
CSL modulates mRNA expression of Nrf2 pathway-related genes in the SHR model and H_2_O_2_-induced conditions. (**a**). Relative mRNA expression levels of Nrf2, Nqo1, Ho-1, and Keap1 across the CON, SHR, L-CSL + SHR, and H-CSL + SHR groups. (**b**–**e**). Relative mRNA expression of Nrf2 (**b**), Nqo1 (**c**), Ho-1 (**d**), and Keap1 (**e**) among the CON, H_2_O_2_, CSL + H_2_O_2_, and SFN + H_2_O_2_ groups. Statistical significance is indicated as follows: * *p* ≤ 0.05, significant; ** *p* ≤ 0.01, highly significant; *** *p* ≤ 0.001, extremely significant; **** *p* ≤ 0.0001, exceptionally significant.

As shown in Figure 7a, the relative mRNA expression levels of Nrf2, Nqo1, and Ho-1 in the kidneys of SHRs were significantly downregulated compared with the CON group (*p* ≤ 0.01), indicating catalase suppression of this pathway in hypertensive kidneys. The relative mRNA expression levels of Keap1 in the kidneys of SHRs were significantly upregulated compared with the CON group (*p* ≤ 0.001). In both L-CSL groups, the mRNA levels of Nrf2 and Nqo1 were significantly higher than those in the SHR group (*p* ≤ 0.01), with a highly significant difference between the two CSL doses (*p* ≤ 0.01). In both L-CSL and H-CSL groups, the mRNA levels of Keap1 were significantly lower than those in the SHR group (*p* ≤ 0.01). These findings suggest that CSL exerts a therapeutic effect by modulating the Nrf2/Ho-1 pathway in the kidneys of SHRs, with this effect being dose-dependent.

As shown in Figure 7b, treatment with H_2_O_2_ significantly altered the mRNA expression of Nrf2 pathway-related genes: relative mRNA levels of Nrf2, Nqo1, and Ho-1 were markedly decreased; in comparison, Keap1 mRNA expression was notably increased (*p* ≤ 0.001 or *p* ≤ 0.0001). Pretreatment with CSL reversed the H_2_O_2_-induced abnormal gene expression: mRNA levels of Nrf2, Nqo1, and Ho-1 were significantly upregulated (*p* ≤ 0.01 or *p* ≤ 0.05), and Keap1 mRNA was downregulated (*p* ≤ 0.01), with effects comparable to those of the positive control SFN.

### 2.8. Molecular Docking

**Figure 8:**
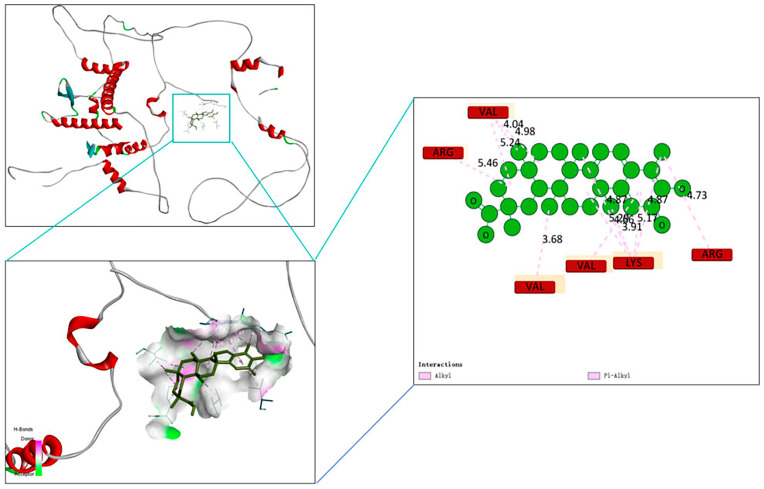
Molecular docking binding mode analysis of the compound with the target protein. The top-left panel shows the 3D structure of the target protein (red helices represent α-helices), and the boxed area indicates the binding pocket of the compound; the bottom-left panel displays the docking conformation of the compound (black backbone) within the binding pocket (the green region represents the protein binding interface); the right panel illustrates the interactions between amino acid residues (labeled in red/yellow) in the binding pocket and the compound (green spheres). Pink dashed lines denote Pi-alkyl interactions, and the values correspond to the distance (Å) between residues and the compound.

A binding energy lower than 0 indicates spontaneous binding between two molecules, and the smaller the binding energy, the more stable the conformation. The molecular docking results presented in Figure 8 indicate that the binding energy between CSL and Nrf2 is −32.2168 k·J/mol, indicating that CSL can exhibit a certain binding affinity for Nrf2.

## 3. Discussion

The results of previous studies have demonstrated that CSL attenuates renal ischemia–reperfusion injury (IRI) via NF-κB inhibition [22]. However, in this study, we focus on its antioxidant activity mediated by the Nrf2/Ho-1 pathway—a mechanism that remains relatively understudied in hypertensive renal damage. Based on the established finding that oxidative stress and inflammation are key drivers of hypertensive kidney injury [2,23], our work is the first case in which the molecular mechanisms underlying CSL’s renal protective effects in SHRs are elucidated.

Hypertensive renal injury is characterized by endothelial dysfunction, inflammatory cell infiltration, and impaired renal regulation of blood pressure via renin secretion and fluid balance [24]. From a histopathological perspective, SHR kidney tissues exhibit significantly increased inflammatory cell infiltration (Figure 7a), with a corresponding inflammatory infiltration score markedly higher than that of the healthy control group (Figure 7b), directly reflecting the inflammatory phenotype of hypertensive renal injury. Following CSL intervention, the degree of inflammatory infiltration is dose-dependently reduced, further verifying its anti-inflammatory effects at the histological level. The results of previous studies have confirmed that these pathological processes are accompanied by abnormal expression of renal biomarkers, including renin, aldosterone, angiotensin II, angiotensin-converting enzyme, and creatinine [6,25]. Consistent with these reports, our findings demonstrate that CSL reverses the abnormal levels of these markers, alleviates pathological damage, and links systemic hypertension control to local renal protection (Figure 1). Persistent hypertension induces renal oxidative stress and inflammation, which synergistically enhance angiotensin-converting enzyme activity and exacerbate kidney damage [26]. The accumulation of inflammatory cytokines further promotes renal fibrosis [27], highlighting the importance of anti-inflammatory interventions in slowing the progression of hypertensive renal injury. Natural compounds have shown promising therapeutic potential in treating renal fibrosis; as an active component of *Tripterygium wilfordii* (clinically used for immunological disorders) [28], CSL reduces renal inflammation by upregulating IL-10 and downregulating tumor necrosis factor-α [29], interleukin-1β, and IL-18 [13]. Consistent with these findings, our results confirm CSL’s anti-inflammatory effects in hypertensive renal injury, supporting the feasibility of anti-inflammatory strategies as potential therapeutic approaches for this disease (Figure 4a).

From concentration screening results, CSL + SFN showed no significant effect on RGEC viability, with high doses inhibiting proliferation; thus, safe concentrations were selected for subsequent experiments (Figure 5). Molecular docking indicated stable binding between CSL and Nrf2 (Figure 8). Combined with gene- and protein-level data, CSL reversed H_2_O_2_-induced suppression of the Nrf2 pathway: upregulating Nrf2, Nqo1, and Ho-1 and downregulating Keap1, with effects comparable to SFN (Figure 7b–e). These findings suggest that CSL may exert antioxidant protection in renal cells by activating the Nrf2 pathway.

Furthermore, while the authors of previous studies have reported that CSL exerts vascular antioxidant effects via Ho-1 upregulation [30], our findings extend this effect to renal tissues: CSL enhances the activity of antioxidant enzymes (superoxide dismutase, glutathione peroxidase, and catalase) and reduces the level of the oxidative damage product malondialdehyde in SHR kidneys. This effect with Younis et al.’s finding that CSL reduces malondialdehyde and increases glutathione (GSH) levels [22], filling a crucial gap in hypertensive nephropathy research (Figure 3). Excessive reactive oxygen species (ROS) production in SHRs induces renal cell damage and apoptosis [31]; however, after dissociating from Keap1, Nrf2 counteracts this damage by activating antioxidant genes (e.g., superoxide dismutase and Ho-1), mitigating injury caused by ischemia–reperfusion or toxins [32]. As a critical cytoprotective enzyme, Ho-1 converts pro-oxidant heme into biliverdin/bilirubin and carbon monoxide (CO)—molecules with antioxidant, anti-apoptotic, and vasodilatory properties [33].

In terms of pathway molecule expression, both protein expression (Figure 7a,c) and mRNA levels (Figure 8) of Nrf2 are compensatorily upregulated in SHR kidneys, whereas Keap1 expression shows a downward trend (Figure 7a,d and Figure 8), representing a defensive response to injury, consistent with previous reports [34]. Following CSL intervention, mRNA expression of Nrf2, Ho-1, and the downstream target gene Nqo1 is further increased (Figure 8), and Keap1 expression continues to decrease, suggesting that CSL amplifies Nrf2/Keap1 pathway activation. Additionally, Nrf2 nuclear translocation (Figure 6e) is significantly increased after CSL intervention, whereas cytoplasmic Keap1 expression (Figure 6f) is correspondingly downregulated. This result directly confirms that CSL promotes Nrf2 dissociation from Keap1 and subsequent nuclear translocation, thereby activating transcription of downstream antioxidant and anti-inflammatory genes—a core molecular event underlying its renal protective effects (Figure 6 and Figure 7).

Despite these insights, several limitations remain in the present study. The absence of an untreated normotensive control group prevents comparison of baseline renal function between hypertensive and normotensive states, limiting conclusions about the hypertension-specific effects of CSL on renal injury. Additionally, we did not perform long-term toxicity assessments, preventing us from clarifying the potential impacts of prolonged CSL administration on liver function, renal function, the hematological system, and other aspects, thereby restricting an accurate evaluation of its clinical safety. In clinical settings, patients with hypertensive renal injury often experience progressive renal function decline and multi-target pathological changes. Considering that CSL, as a natural compound, possesses pleiotropic anti-inflammatory and antioxidant activities, the authors of future preclinical studies could explore its synergistic effects with clinically commonly used antihypertensive drugs to enhance its enrichment in renal tissues, thus providing a more targeted candidate regimen for combination therapy of hypertensive nephropathy. 

## 4. Materials and Methods

### 4.1. Animal

To minimize gender-related variability, only male rats were used in this study. The experimental animals were spontaneously hypertensive rats (SHRs) (6–8 weeks old, 220–250 g, purchased from Beijing, License No. SCXK [Beijing] 2016-0006), with age-matched normotensive Wistar–Kyoto (WKY) rats as the healthy control group. Rats were housed in a specific pathogen-free (SPF) facility under controlled environmental conditions, including a 12 h light/dark cycle, 40% relative humidity, and a constant temperature of 25 °C. Four rats were housed per cage. A total of 40 rats were randomly divided into four groups (*n* = 8 per group) using a random number table to minimize selection bias: control (CON, rats as the healthy control), spontaneously hypertensive rats (SHRs), SHRs with low-dose CSL (L-CSL + SHR, 0.5 mg/kg/d), and SHRs with high-dose CSL (H-CSL + SHR, 1 mg/kg/d). After completing the overall phenotypic assessment, stratified random sampling was performed based on baseline indicators such as body weight and organ indices, with tissue samples from 3 animals selected for subsequent molecular mechanism detection. All statistical analyses were conducted using these 3 independent biological replicates.

### 4.2. Specific Protocol

CSL injection solutions were prepared using physiological saline as the solvent. Rats in the CON and SHR groups received intraperitoneal injections of an equivalent volume of physiological saline. Rats in the L-CSL + SHR and H-CSL + SHR groups received intraperitoneal injections of CSL at a dose of 0.5 mg/kg/d and 1 mg/kg/day. All intraperitoneal injections were administered once daily for a continuous 6-week treatment period. At the end of the intervention period, all rats were euthanized, and blood samples and kidney tissue specimens were collected for subsequent assays. In detail, blood analysis was performed to determine the serum levels of renin, angiotensin, angiotensin-converting enzyme, and aldosterone, combined with the concentrations of inflammatory factors including tumor necrosis factor-α, interleukin-1β and interleukin-6. Kidney tissue specimens were subjected to antioxidant index detection (to measure the levels of malondialdehyde, superoxide dismutase, glutathione peroxidase and catalase), histological examination and immunohistochemistry (to observe renal pathological morphological changes and localize target proteins), Western blotting analysis (to detect the protein expression levels of Keap1, Nrf2, Nqo1, and Ho-1), and real-time PCR (to assess the mRNA expression levels of Nrf2, Nqo1, and Ho-1).

### 4.3. Randomization

To perform sample collection and subsequent experiments, a randomization procedure and single-blind design were implemented to ensure objective results. Randomization was performed prior to grouping: rats were randomly assigned to experimental groups using a random number table generated with GraphPad Prism 9.5 software, and each group was labeled with a unique code to conceal group identities during the experiment. A single-blind procedure was applied as follows: the researcher responsible for animal sampling and grouping was aware of the group assignments only for operational purposes; analysts involved in subsequent serum biochemical tests, histopathological examinations, immunohistochemical staining and fluorescence intensity quantification, and Western blot experiments were blinded to the group information—they only received samples labeled with unique codes and were unaware of the corresponding group assignments until all experimental data were collected and recorded. Rats were anesthetized with 5% isoflurane (R510-22-10, RWD, Shenzhen, China), and blood samples were collected from each experimental group. Serum was obtained by means of centrifugation at 5000 rpm for 15 min and stored at −20 °C for further analysis. The right kidney was rapidly excised and divided into two portions: one portion was homogenized and stored at −80 °C for biochemical assays, and the other was fixed in 4% paraformaldehyde (E672001-5000, Sangon, Shanghai, China) for histopathological testing.

### 4.4. Sample Size

Each group comprised 8 male rats (*n* = 8), serving as biological replicates, to minimize gender-related variability. However, the primary data presented were derived from the initial set of eight rats per group. For each tissue homogenate and serum sample, biochemical assays were conducted in duplicate (technical replication) to ensure precision. Formalin-fixed kidney tissues were randomly sectioned into five slices per sample, with three non-overlapping fields of view examined under a microscope for each section. Histopathological scoring, including the assessment of inflammatory infiltration, was performed by a pathologist unaware of the group assignments. The mean score across all fields and sections was utilized for statistical analysis to mitigate the impact of individual variability.

## 5. Experimental Design

### 5.1. Blood Analysis

Serum samples were thawed at 4 °C and then thoroughly mixed by means of gentle vortexing. Using a previously described protocol, prior to serum separation, the experimental rats were anesthetized with isoflurane (induction concentration: 2%; maintenance concentration: 1.5%). After 12 h of fasting, abdominal aortic blood samples were collected between 9:00 and 10:00 a.m. Throughout the experiment, all rats were fed a standard rodent diet containing 0.3% sodium. The whole blood was placed in serum separation tubes without anticoagulants, allowed to clot at room temperature for 30 min, and then centrifuged at 3000× *g* for 15 min at 4 °C within 1 h of blood collection. No protease inhibitors were used during blood processing, because the commercial enzyme-linked immunosorbent assay (ELISA) kits employed in this experiment had all been validated and were suitable for direct detection of serum samples. A 300 μL aliquot of each serum sample was added to individual wells, with three technical replicates established for each sample. Subsequently, the samples were diluted at a ratio of 1:5 using the dilution buffer provided with the kits. Concentrations of renin (E-EL-R0030c, Elabscience, Wuhan, China), angiotensin II (RE2892R, Reed, Wuhan, China), angiotensin-converting enzyme (E-EL-R2401c, Elabscience, Wuhan, China), aldosterone (E-EL-0070c, Elabscience, Wuhan, China), and creatinine (ZK-6963, Chemical Book, Shanghai, China) were determined in strict accordance with the manufacturers’ instructions for each respective kit. Additionally, concentrations of tumor necrosis factor-α (TNF-α, E-HSEL-R0001, Elabscience, Wuhan, China), interleukin-1β (IL-1β, E-EL-R0012, Elabscience, Wuhan, China), and interleukin-6 (IL-6, 900-K86, Thermo Fisher, Waltham, MA, USA) were measured following the detection protocols of their corresponding kits. To eliminate variations in protein content among different samples and ensure data reliability, all measured values were normalized to serum protein concentration. The rationale for normalizing hormone levels to total protein is to eliminate potential variations in serum sample volume or concentration caused by differences in blood collection volume, hemolysis degree, or processing procedures among individual rats, thereby ensuring the accuracy and comparability of the detected hormone concentrations.

### 5.2. Antioxidant Indices

Renal levels of superoxide dismutase (SOD; catalog no. A001-1-2), glutathione peroxidase (GSH-Px; catalog no. A005-1), catalase (CAT; catalog no. A007-1), and malondialdehyde (MDA; catalog no. A003-1) were determined by means of spectrophotometry using a PU 8720 UV/VIS scanning spectrophotometer (Persee Instruments, Beijing, China). Commercial assay kits were purchased from the Nanjing Jiancheng Institute of Bioengineering (Nanjing, China), and all measurements were performed strictly in accordance with the manufacturers’ protocols. The enzyme activities of SOD, GSH-Px, and CAT are expressed as U/mg protein, whereas the concentration of MDA is expressed as nmol/mg protein. All results were normalized to the total renal tissue protein concentration to eliminate variations caused by differences in sample loading.

### 5.3. Histology Examination and Immunohistochemistry

Kidney samples were collected and fixed in 4% paraformaldehyde at 4 °C overnight. To perform subsequent procedures, including paraffin embedding, sectioning, and staining, standard histological techniques were followed as detailed in our previous publication [35]. The histological scoring criteria for renal tissue damage were defined as follows: 0 points = no detectable damage, 1 point = damaged area < 10%, 2 points = damaged area 10–25%, 3 points = damaged area 26–50%, 4 points = damaged area 50–75%, and 5 points = damaged area > 75% [36].

To perform immunohistochemistry, the kidney tissues were embedded in Tissue-Tek OCT and snap-frozen in liquid nitrogen. Subsequently, the samples were cryosectioned at a thickness of 15 µm and stained for the target proteins. To ensure staining specificity, control groups were included: Negative controls: sections were incubated with phosphate-buffered saline (PBS, pH 7.4) in place of primary antibodies; Isotype controls: sections were incubated with a rabbit IgG isotype control antibody (ab172730, Abcam, Cambridge, UK) at the same concentrations (1:300 for Nrf2, 1:200 for Keap1) as the primary antibodies to exclude non-specific binding. Antibodies raised against the following rat antigens were utilized: an Nrf2 antibody at a dilution of 1:300 (ab137550, Abcam, Cambridge, UK) and a Keap1 antibody at a dilution of 1:200 (ab218815, Abcam, Cambridge, UK). Consequently, a species-matched secondary antibody was employed to amplify the signals: Alexa Fluor 488 anti-rabbit IgG (ab150077, Abcam, Cambridge, UK, dilution 1:500). Nuclei were counterstained with Hoechst 33,342 (H3570, Thermo Fisher, Waltham, MA, USA; 10 µg/mL for 10 min at room temperature). Two slides from each rat (*n* = 3 per group) were imaged using a Zeiss confocal laser scanning microscope (Carl Zeiss Microscopy GmbH, Jena, Germany), model 780, with consistent laser power, gain, and exposure settings across all samples. Fluorescence intensity was quantified using ImageJ 1.8.0 software (National Institutes of Health, Bethesda, MD, USA): three non-overlapping fields of view were randomly selected per section, the mean fluorescence intensity (MFI) of the target protein (Nrf2/Keap1) was measured in the renal cortical region, and background intensity was subtracted using adjacent non-stained areas. The average MFI across all fields and slides per rat (*n* = 3 per group) was used for statistical analysis.

### 5.4. Western Blotting Analysis

The mixture was used to homogenize kidney tissue after adding phosphatase and protease inhibitors (1% *v*/*v* each) to cell lysis buffer (BL509A, Biosharp, Hefei, China). Protein concentrations of the lysates were determined using a BCA assay (562 nm, bovine serum albumin standard), and samples were adjusted to a uniform concentration of 2 mg/mL. Next 20 μg of total protein per lane was loaded for separation on 10% SDS-PAGE gels. Proteins were transferred to PVDF membranes (IPVH00010, Millipore, Burlington, MA, USA) and blocked for 12 h with 5% skim milk. Membranes were then incubated separately with primary antibodies (Nrf2: ab137550, Abcam, Cambridge, UK, 1:1000, 66 kDa) and the housekeeping protein antibody (β-actin: ab8226, Abcam, Cambridge, UK; 1:2000, 42 kDa) for 1 h, followed by incubation with a species-matched secondary antibody (ab150077, Abcam, Cambridge, UK) for 1 h. Lastly, membranes were scanned and analyzed using an ODYSSEY two-color infrared laser imaging system (DLX-3421, LI-COR Biosciences, Lincoln, NE, USA). Densitometric analysis of protein bands was performed using ImageJ 1.8.0 software. Regarding Western blot results, the intensity of target protein bands (Nrf2/Keap1) was normalized to the corresponding β-actin band intensity, and the final data were expressed as fold changes relative to the CON group. Equal protein loading was verified by consistent β-actin band intensities across all lanes.

### 5.5. Real-Time PCR

The kidney tissues from rats were utilized for RNA isolation using the Trizol TRIzol™ Plus RNA Purification Kit (12183555, Invitrogen, Carlsbad, CA, USA). The optical density of RNA was calculated at 2.066, stored at −80 °C. Subsequently, the RNA was reverse-transcribed into cDNA according to the provided instructions (R312-01, Vazyme, Nanjing, China) and mRNA expression levels were determined in accordance with the manufacturer’s protocol (Q231, Vazyme, Nanjing, China) (the primers are listed in Table 1). Amplification efficiency was validated, and relative expression was calculated using the 2^−ΔΔCT^ method [37].

### 5.6. Cell Grouping

The Rat Glomerular Endothelial Cell (RGEC) line was cultured in DMEM medium containing 15% fetal bovine serum, 100 U/mL penicillin, and 100 mg/L streptomycin at 37 °C in a 5% CO_2_ incubator. The RGECs were divided into the CON group, H_2_O_2_ group, CSL + H_2_O_2_ group, and Nrf2 agonist + H_2_O_2_ group (SFN + H_2_O_2_). The CON group was supplemented with 2 mL of serum-free medium; the CSL + H_2_O_2_ group received 2 mL of 3 μM CSL pretreatment for 24 h; the SFN + H_2_O_2_ group received 2 mL of 2 μM SFN pretreatment for 24 h. Excluding the CON group, all groups were treated with 800μmol/L H_2_O_2_ for 24 h [38].

### 5.7. RGEC Culture

RGECs were cultured in DMEM medium supplemented with 100 mL/L fetal bovine serum and 10 mL/L penicillin/streptomycin in a 37 °C incubator with 50 mL/L CO_2_. When the cells reached the logarithmic growth phase, they were seeded at 4 × 10^4^ cells per well in a 96-well plate. The cells were treated with 800 μmol/L H_2_O_2_, simulating renal oxidative stress injury in this study. RGECs were seeded at 4 × 10^4^ cells per well in a 96-well plate and treated with CSL concentrations of 1, 3, 6, 12, 24, and 48 μmol/L for 24 h, in addition to SFN concentrations of 1, 2, 4, 8, 16, and 32 μmol/L for 24 h. The concentrations of CSL and SFN were screened according to the CCK-8 assay instructions (C0038, Beyotime, Shanghai, China). The OD 450 values were measured to determine the maximum safe concentrations of AZM and PF.

### 5.8. Molecular Docking

The 3D structure of CSL was downloaded from the PubChem database (https://pubchem.ncbi.nlm.nih.gov/). The structure of Nrf2 was obtained from the RCSB Protein Database (https://www.rcsb.org/, URL (accessed on 1 January 2026). Docking simulations were performed using Autodock Vina; 20 runs were conducted, and the CSL-Nrf2 complex with the optimal binding energy was selected. Lastly, the results were visualized using Pymol 4.0 software.

### 5.9. Statistical Analysis

All data are expressed as mean ± standard deviation, and SPSS 28.0.1.1 statistical software was used for single-factor variance analysis (ANOVA). Duncan’s multiple-range test was used for comparison. All data were found to conform to a normal distribution. GraphPad Prism 9.5 software was used for plotting the results. The statistical significance is indicated as follows: * *p* ≤ 0.05, significant; ** *p* ≤ 0.01, highly significant; *** *p* ≤ 0.001, extremely significant; **** *p* ≤ 0.0001, exceptionally significant.

## Figures and Tables

**Table 1:** Nrf2, Ho-1, Nqo1, and Keap1 oligonucleotide primers used for qRT-PCR analysis.

Gene	Sequence (5′ Approximately 3′)
Nrf2	F-5′-GACCTAAAGCACAGCCAACACAT-3′
	R-5′-CTCAATCGGCTTGAATGTTTGTC-3′
Ho-1	F-5′TGTCCCAGGATTTGTCCGAG-3′
	R-5′-ACTGGGTTCTGCTTGTTTCGCT-3′
Nqo1	F-5′-GGGGACATGAACGTCATTCTCT-3′
	R-5′-AGTGGTGACTCCTCCCAGACAG-3′
Keap1	F-5′-ATGTGATGAACGGGGCAGTC-3′
	R-5′-AGAACTCCTCCTCCCCGAAG-3′

## Data Availability

The original contributions presented in the study are included in the article. Further inquiries can be directed to the corresponding author.
